# Analysis of Passivation and Corrosion Processes of Modified LaNi_5_ Alloy-Based Hydride Electrodes

**DOI:** 10.3390/ma19102076

**Published:** 2026-05-15

**Authors:** Krystyna Giza, Edyta Owczarek, Joanna Piotrowska-Woroniak, Grzegorz Woroniak

**Affiliations:** 1 Faculty of Production Engineering and Materials Technology, Czestochowa University of Technology, 19 Armii Krajowej Ave., 42-200 Czestochowa, Poland; krystyna.giza@pcz.pl; 2 Department of Water Supply, Sewerage and District Heating, Bialystok University of Technology, 45E Wiejska St., 15-351 Bialystok, Poland; j.piotrowska@pb.edu.pl (J.P.-W.); g.woroniak@pb.edu.pl (G.W.)

**Keywords:** AB_5_ hydrogen storage alloys, passivation, corrosion resistance

## Abstract

Studies were conducted on the effect of the partial substitution of nickel in an LaNi_5_ alloy with germanium (5% by weight) or magnesium (3.3% by weight), in addition to surface modification using phosphomolybdic heteropolyacid (MPA) on the course of corrosion and passivation processes of hydrogen electrodes in a highly alkaline environment. The investigations were carried out by means of electrochemical impedance spectroscopy (EIS) and the potentiodynamic methods to analyse changes in the electrochemical parameters as a function of exposure time. The surface topography of the electrodes and chemical composition were investigated utilising a KEYENCE VHX-7000 digital microscope (Osaka, Japan) and a scanning electron microscope (SEM) equipped with an energy-dispersive spectroscopy EDS X-ray microanalysis attachment. The novelty of this work lies in the systematic, time-dependent comparison of the effects of bulk and surface modifications on the evolution of corrosion-passivation mechanisms of electrodes based on the LaNi_5_ alloy. It has been shown that the Mg and Ge additives improve corrosion resistance in the initial stage of exposure but lead to destabilisation of the passive layer during prolonged electrolyte interaction. A different effect was observed for the MPA-modified electrodes, in which a stable protective layer forms, limiting corrosion while maintaining favourable hydrogen desorption kinetics. The obtained results indicate the key role of exposure time (>140 h) in shaping the corrosion mechanisms and emphasise the need for simultaneous optimisation of the alloy composition and surface properties in the design of durable hydrogen electrodes.

## 1. Introduction

Despite the rapid development of lithium-ion battery technology, nickel-metal hydride (Ni-MH) batteries have remained a key component of energy storage systems for many years, particularly in hybrid vehicles, industrial installations and uninterruptible power supplies [1,2]. Their popularity stems from their high operational reliability, thermal stability and environmental friendliness compared to lithium-ion cells [3]. The performance parameters and service life of Ni–MH batteries are largely determined by the properties of the negative electrode [3], which is usually manufactured on the basis of AB_5_-type intermetallic compounds with a CaCu_5_-type structure, capable of reversibly binding hydrogen within the crystal structure of the material under low pressure [4].

Among these, LaNi_5_ alloys are of particular significance; they are widely used commercially due to their stable thermodynamic properties, low equilibrium pressure [5] and good kinetics of hydrogen absorption/desorption processes [6]. However, studies indicate that LaNi_5_ hydride electrodes, despite their favourable electrochemical and structural properties, are susceptible to both corrosion [7,8] and mechanical degradation under long-term operating conditions, which is a significant limitation to their practical application [9,10]. Repeated hydrogen charging/discharging cycles are accompanied by cyclic changes in the crystal lattice parameters, leading to the generation of internal stresses in the anode material [9]. As the number of cycles increases, these stresses promote the formation of microcracks, particle fragmentation and the gradual pulverisation of the alloy, leading to a rapid decline in the hydrogen storage capacity, even below 50% [11,12].

In parallel with mechanical degradation, the surface of the anode, when subjected to continuous exposure in a strongly alkaline KOH solution, is exposed to corrosive processes leading to deactivation of the material [11,12]. The authors of numerous studies have confirmed that in an alkaline environment, processes of selective oxidation and dissolution of the components of MmNi_5_-type alloys take place, in particular Mm [7,8]. As a result of these processes, a layer of reaction products forms on the electrode surface, consisting mainly of acicular Mm(OH)_3_ crystals [7,13,14], which acts as a barrier impairing the kinetics of hydrogen adsorption/desorption processes [8]. The transfer of lanthanum into the solution in the form of La^3+^ ions leads to gradual enrichment of the surface with nickel, which may initially increase the catalytic activity of the anode; nonetheless, over time, this promotes structural degradation and a decrease in its capacity during repeated cycling, and consequently shortens the service life of the cell [8,9]. These phenomena are directly related to passivation processes occurring in strongly alkaline media, where the surface layer is continuously formed, transformed, and partially dissolved during electrode operation. Therefore, the stability and composition of this layer strongly determine both corrosion resistance and electrochemical activity of LaNi_5_-based electrodes. In this context, modification of the alloy composition and surface properties represents an effective approach to controlling passivation behaviour.

A widely used method for improving the properties of LaNi_5_ alloys is the partial substitution of nickel with other elements. Numerous studies have shown that alloying elements such as Cu [15], Co [16], Al [17], Mn [18,19], Sn [20], Ge [21,22,23], as well as Mg, Bi, and Sb [13,24] significantly influence the crystal lattice parameters, hydrogen sorption properties, and the corrosion resistance of the material.

As demonstrated in previous studies on solid LaNi_5−x_Ge_x_ alloys [21], an increased germanium content (approx. 10% by weight) in the LaNi_5_ alloy leads to a significant reduction in electrode surface degradation. At the same time, a reduction in the hydrogen capacity and a deterioration in the charge transport kinetics at the electrode/electrolyte interface are observed [21]. Reducing the germanium content to around 5% by weight has a beneficial effect on the charge transfer process at this interface [21].

In [13], we demonstrated the positive effect of magnesium on the anti-corrosion properties of the electrode materials under investigation following prolonged exposure to an alkaline electrolyte solution. The authors of other studies [24] emphasise that the presence of a small amount of Mg improves the hydrogenation kinetics of LaNi_5_-type alloys as well as their hydrogen storage capacity.

At the same time, surface modification strategies are being developed, which serve as an effective tool for limiting the degradation of electrode materials. The physicochemical properties of the passive layer formed at the electrode/electrolyte interface play a key role in controlling the kinetics of corrosion processes and the course of electrochemical reactions [25,26,27]. Phosphomolybdic acid, in particular, demonstrates the ability to modify surfaces, facilitate charge transfer processes and stabilise the passive layer, which results in a reduction in corrosion rates [26,27].

Despite the numerous studies on the modification of LaNi_5_ alloys, most of the work to date has focused on single strategies for improving the material properties, such as partial elemental substitution or surface modification, which are usually analysed independently. Hence, there is still a lack of systematic comparative studies considering the simultaneous impact of these approaches on passivation and corrosion mechanisms, especially under long-term exposure to a highly alkaline environment. The novelty of this work lies in the integrated analysis of the effect of bulk (Mg, Ge) and surface (MPA) modifications on the electrochemical properties as well as corrosion-passive mechanisms of LaNi_5_ alloy-based electrodes. Particular attention was paid to the changes in these properties as a function of exposure time, which enabled the identification of distinct trends in passive layer stability, in addition to their impact on the corrosion rate and hydrogen kinetics.

The aim of this study is to conduct a comprehensive analysis of the effect of a partial substitution of nickel with germanium (5 wt%) or magnesium (3.3 wt%) and surface modification using phosphomolybdenum heteropolyacid (MPA) on the corrosion mechanisms, passivation processes and electrochemical properties of LaNi_5_-based composite hydride electrodes in a strongly alkaline environment.

## 2. Experimental Section

### 2.1. Experimental Materials

The alloys were prepared by arc melting stoichiometric amounts of elements (99.99% purity, Sigma Aldrich, St. Louis, MO, USA) in a protective argon atmosphere. After melting, the alloy samples were homogenised at 600 °C for 300 h to ensure chemical homogeneity while minimising magnesium evaporation (germanium is stable under these conditions) and preserving phase stability. The alloys were remelted under an argon atmosphere to ensure homogeneity. All the samples were prepared under identical conditions to ensure reproducibility. To prevent magnesium losses during synthesis, a slight excess of this metal, about 5%, was used relative to the intended stoichiometry. For comparison purposes, a commercial LaNi_5_ alloy with a purity of 99.9% (Alfa Aesar GmbH & Co., KG, Karlsruhe, Germany) was used.

To determine the phase composition of the obtained alloys, diffraction studies were performed using a Bruker D8 Advance diffractometer (Karlsruhe, Germany) equipped with a Johansson monochromator (λCu K_α_1 = 1.5406 Å) and a LYNXEYE strip detector (Bruker AXS GmbH, Karlsruhe, Germany).

Three types of composite powder electrodes were prepared, namely based on LaNi_5_, LaNi_4.8_Mg_0.2_, and LaNi_4.7_Ge_0.3_ alloys, by mixing 85 wt% of the active material having a particle size of 20–50 µm with C45 carbon black in an amount of 5 wt%. Electrodes were also prepared using 85 wt% LaNi_5_ and 5 wt% C45 carbon black modified with phosphomolybdenum heteropolyacid (LaNi_5_-MPA electrode). Polyvinylidene fluoride (PVDF) was used as a binder at 10% by weight. A detailed description of the alloy preparation process, electrode fabrication, and the modification of C45 carbon black with heteropolyacid can be found in our previous publications [13,21,27].

### 2.2. Experimental Methodology

Electrochemical studies were carried out on composite powder electrodes containing 0.030–0.035 g of hydrogen storage alloy, employing direct and alternating current techniques in a three-electrode system. The auxiliary electrode was a platinum electrode, and the reference electrode was a saturated calomel electrode (SCE). All the measurements were performed in a 6 M sodium hydroxide solution at room temperature by means of a CHI 760 B measuring device from CH Instruments, Inc. (Austin, TX, USA).

Impedance spectra were generated for each electrode after 24, 50, 74, 142, 214 and 243 h of exposure in a 6 M NaOH solution (pH = 15.2), respectively, in the frequency range from 100 kHz to 0.01 Hz at open-circuit potential (OCP). Potentiokinetic polarisation curves were recorded at identical time intervals within a potential range of ±0.2 V relative to OCP at a scan rate of 0.001 V s^−1^. Due to the passivation of the alloy in the alkaline electrolyte, the corrosion current density was calculated by extrapolating the cathodic Tafel branch to *E*_corr_. A Keyence VHX-7000 microscope (Osaka, Japan) was used to measure 3D roughness parameters before and after prolonged exposure of the electrodes in 6 M NaOH, as well as to observe the surface morphology. Analysis of the chemical composition of the composite powder electrode surfaces was performed by utilising a JEOL JSM-6610 LV (Tokyo, Japan) scanning electron microscope (SEM) coupled with an energy-dispersive X-ray spectrometer (EDS).

## 3. Results and Discussion

### 3.1. X-Ray Structural Analysis

**Figure 1:**
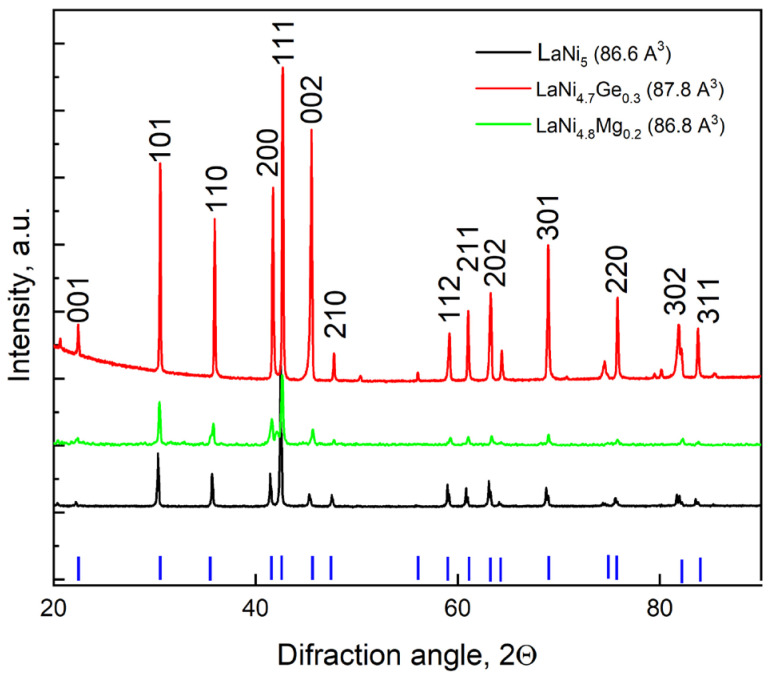
X-ray diffraction patterns of LaNi_5−_xM_x_ (M = Ge, Mg) alloys and the theoretical diffraction pattern of the LaNi_5_ compound taken from the Pearson Crystal Data: Crystal Structure Database for Inorganic Compounds [28]. Blue tick marks indicate the reference diffraction peak positions of the LaNi_5_ phase.

The results of the structural analysis shown in Figure 1 indicate that the LaNi_5−x_M_x_ alloy samples obtained by partially substituting nickel in the LaNi_5_ intermetallic compound with magnesium to a composition of x = 0.2 or with germanium to a composition of x = 0.3 retain the crystalline structure of the starting material (a CaCu_5_-type hexagonal structure). These materials are single-phase with parameters corresponding to the LaNi_5_ phase [28].

Using Rietveld analysis software, (FullProf Suite, Institut Laue-Langevin, Grenoble, France, available online: https://www.ill.eu/sites/fullprof/, accessed on 14 May 2025) the lattice parameters for the individual alloys were calculated, and the unit cell volumes were determined on this basis. Although peak shifts in Figure 1 are slight, the numerical values of the unit cell volume (determined via Rietveld analysis) clearly demonstrate the influence of Mg and Ge substitution on the lattice parameters. As can be seen from Figure 1, the unit cell volume undergoes slight changes as a result of the partial substitution of nickel in the LaNi_5_ alloy with magnesium, and grows slightly upon substitution with germanium. A larger unit cell volume correlates with an increase in interstitial spaces that can accommodate more hydrogen atoms, and with a drop in the equilibrium pressure of hydrogen since these atoms are less tightly bound in the expanded crystal lattice, which is extremely beneficial for the application of such materials in Ni-MH cells. Although the unit cell volume remains virtually unchanged when nickel is substituted with magnesium, replacing nickel with magnesium can significantly alter the properties of this alloy, such as the hydrogen storage capacity or stability, as the corrosion of magnesium and its alloys is accompanied by hydrogen evolution, and the rate of this reaction grows with increasing anodic polarisation [29].

### 3.2. Currentless and Steady-State Tests

**Figure 2:**
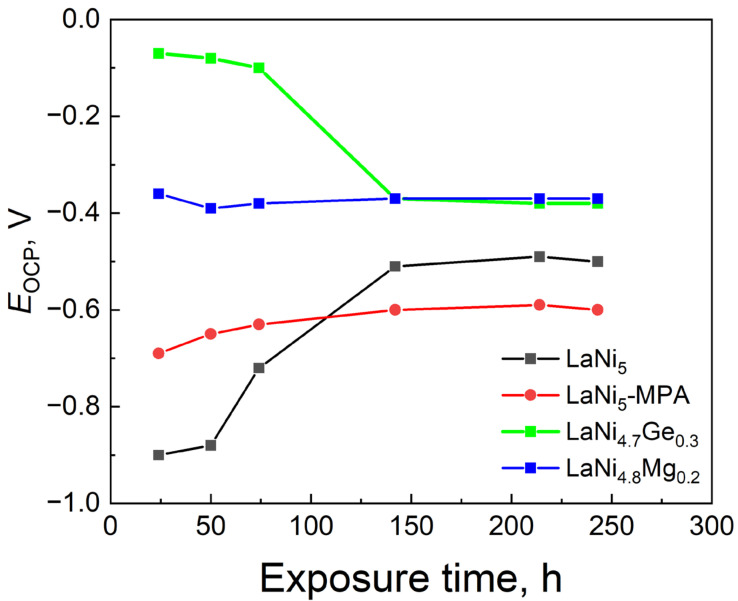
Changes in the stationary potential of studied electrodes as a function of immersion time in 6 M NaOH.

Figure 2 shows the open-circuit potentials (*E*_OCP_) of the composite powder electrodes recorded after 24, 50, 74, 142, 214 and 243 h of exposure in a 6 M NaOH solution. It was only after approximately 140 h of contact between the alloys and the electrolyte that clear stabilisation of the potentials could be observed. The *E*_OCP_ values at that time were, respectively: LaNi_5_: −0.5 V, LaNi_5_-MPA: −0.6 V, LaNi_4.7_Ge_0.3_ and LaNi_4.8_Mg_0.2_: −0.40 V.

Based on the performed *E*_OCP_ measurements, it was found that the initial LaNi_5_ electrode exhibited the greatest susceptibility to corrosion during the initial period of exposure in the electrolyte (approximately 100 h). Nevertheless, after prolonged contact with the solution, a marked rise in the *E*_OCP_ value is observed, indicating the increasing corrosion resistance of the LaNi_5_ alloy. Replacing part of the nickel in LaNi_5_ with either magnesium or germanium appears to be beneficial when considering the thermodynamic susceptibility to corrosion of the materials under investigation.

**Figure 3:**
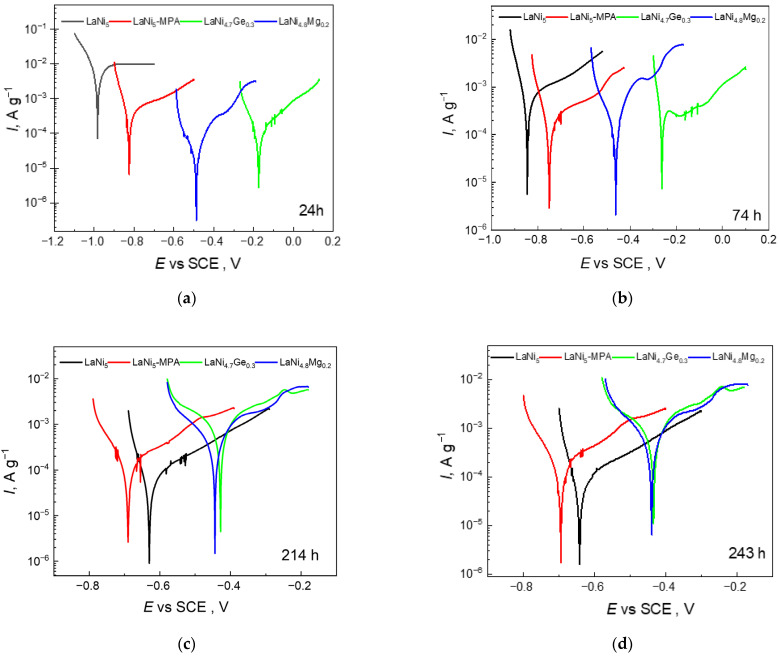
Polarisation curves of investigated electrodes recorded after: (**a**) 24, (**b**) 74, (**c**) 214 and (**d**) 243 h exposition time in 6 M NaOH solution at a scan rate of 1 mV s^−1^.

**Figure 4:**
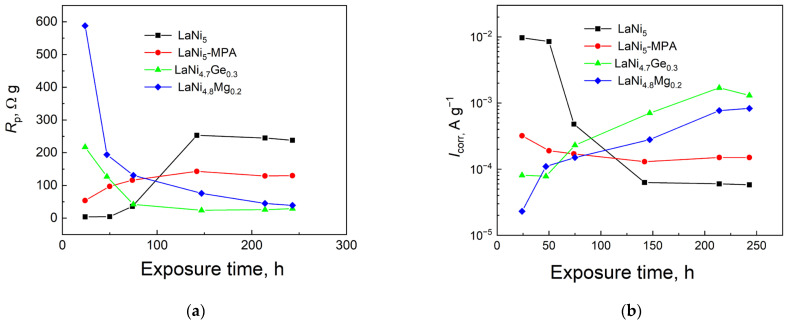
Polarisation resistance (**a**) and corrosion rate (**b**) dependence on exposure time in 6 M KOH solution for investigated electrodes.

However, thermodynamic corrosion susceptibility does not provide any information on the corrosion rate; therefore, the polarisation resistance of the individual electrodes was measured after various exposure times in the 6 M NaOH solution (Figure 3: (a) 24 h, (b) 74 h, (c) 214 h and (d) 243 h). The tests were conducted within a polarisation range of ±0.2 V around the open-circuit potential (OCP). Software utilising the Tafel equation for the calculations was employed to determine the polarisation resistance (*R*_p_) and the corrosion current (*I*_corr_). The obtained values of the parameters *R*_p_ and *I*_corr_ as a function of immersion time in the electrolyte are presented in Figure 4, while their detailed values are summarised in Table 1, which indicates a clear increase in *R*_p_ and a decrease in *I*_corr_ for the LaNi_5_ and LaNi_5_-MPA alloys with increasing exposure time, reflecting improved corrosion resistance, whereas the Mg- and Ge-doped alloys show the opposite trend.

The differences in the polarisation curves for successive measurements taken after varying exposure times of the electrodes in the corrosive solution are likely due to volumetric corrosion resulting from the porosity of the composite powder electrodes. For the first measurement (24 h’ exposure in the solution), the electrode has not yet undergone volumetric oxidation, whereas for subsequent measurements, the porous electrode material undergoes oxidation at significant depths. Furthermore, the electrodes can be further divided into: (1) materials with a more stable passive layer—anodic currents decrease significantly with prolonged contact with the electrolyte (LaNi_5_ reference electrode, LaNi_5_-MPA heteropolyacid-modified electrode) and (2) materials with a more porous, non-sealed oxide layer—anodic currents in the passive range are significantly higher for longer exposure times in the electrolyte (electrodes doped with magnesium and germanium). A common feature of the porous materials in category (2) compared to (1) was the significant shift in the cathode-anode transition potentials towards positive values, which would indicate increased anodic activity in the alloys containing germanium or magnesium.

It is worth noting that the magnesium- and germanium-doped alloys studied in this work exhibit corrosion parameters (*R*_p_, *I*_corr_) that are significantly more favourable than those of the reference alloy (LaNi_5_) for shorter exposure times in the corrosive solution. After 24 h of exposure in the solution, the LaNi_5_ electrode exhibited the lowest polarisation resistance (4.1 Ω g), and thus the highest corrosion rate (0.0097 A g^−1^). Nevertheless, after prolonged contact with the electrolyte (approximately 140 h), the initial corrosion rate of the LaNi_5_ electrode decreases rapidly and is the lowest among the researched electrode materials. This difference is mainly owing to the nature of the passive layer and its stability during exposure to the electrolyte. In the initial stage after immersion of the electrodes in the corrosive solution, lanthanum is mainly dissolved, whilst nickel enriches the surface, leading to its activation. Over time, a passive layer forms on the surface, which stabilises and thickens; consequently, corrosion transitions from active to diffusion-controlled. In the case of the electrodes containing Mg and Ge, the corrosion rate rises over time. In this case, rather than sealing itself, the passive layer undergoes activation through the selective dissolution of Mg or the amphoteric GeO_2_ layer. A reactive surface layer forms, the active surface area grows, as do the kinetics of hydrogen absorption and the corrosion rate.

**Figure 5:**
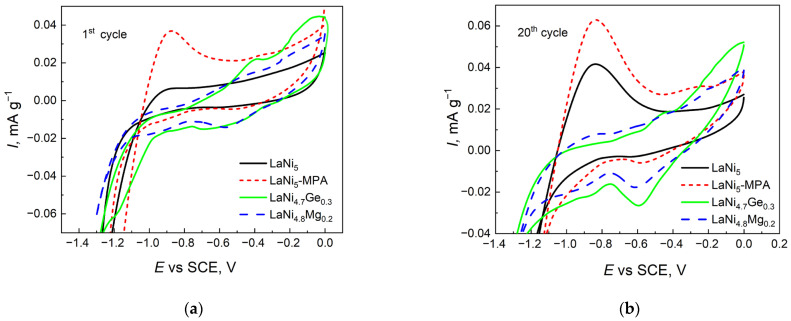
CV curves recorded in 6 M NaOH solution in potential range from 0 to −1.3 V at scanning rate of 10 mV s^−1^ vs. (SCE) for the first (**a**) and twentieth (**b**) cycles.

To assess the influence of the passive layer on the hydrogen absorption/desorption kinetics of the investigated electrodes, cyclic voltammetry (CV) tests were conducted. Figure 5 shows the CV curves of the four composite hydride electrodes after 243 h of exposure in a highly alkaline solution.

Analysis of the cathodic and anodic peaks provides detailed information on the oxidation and reduction processes in the materials under investigation. In Figure 5, only the LaNi_5_ and LaNi_5_-MPA electrodes show a distinct anodic peak at a potential of −0.87 V, which can be attributed to the oxidation of hydrogen accumulated in the alloy. In the case of the alloys containing both germanium and magnesium, this peak is not visible. The corrosion processes of these electrodes cause their surface to be electrochemically ‘open’; hydrogen does not accumulate beneath the passive layer but is immediately oxidised according to the reaction: H_surface_ + OH^−^ → H_2_O + e, which is why the current exhibits a wave-like character. A completely different mechanism of hydrogen absorption/desorption can be observed for the remaining two electrodes. Here, once the hydrogen oxidation potential is reached, hydrogen is released at varying rates. The height of the anodic peak indicates significantly better hydrogen desorption kinetics for the LaNi_5_ electrode modified with the heteropolyacid MPA. Differences in the chemical composition and structure of the passive layer, which forms on the surface of the studied electrodes in contact with the electrolyte, influence surface reactivity. The introduction of magnesium or germanium into the LaNi_5_ crystal lattice alters the thermodynamic characteristics of the surfaces of these materials. The reduction reaction of the passive layers forming on the surface of the LaNi_5_ and LaNi_5_-MPA electrodes is likely too slow or occurs at potentials close to hydrogen evolution, which masks the reduction peak at −0.6 V visible for LaNi_4.7_Ge_0.3_ and LaNi_4.8_Mg_0.2_.

### 3.3. Electrochemical Impedance Spectroscopy (EIS) Test

Nyquist spectra recorded in the high-frequency range allow a more detailed analysis of the surface properties of the passive corrosion layers that form upon contact with the electrolyte.

**Figure 6:**
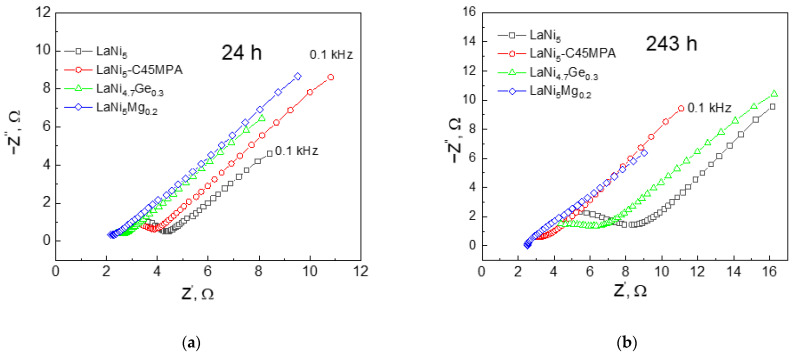
EIS impedance spectra for studied electrodes after (**a**) 24 and (**b**) 243 h of immersion in 6 M NaOH solution in the frequency range of 100–0.1 kHz, amplitude: 5 mV.

The inclination of the spectral bands in Figure 6 (an angle of approximately 45° relative to the horizontal axis) and the absence of a distinct semicircle indicate that the surface of the electrodes is non-uniform and porous in accordance with the transmission line model proposed by de Levie [30]. Nonetheless, the uniform angle of inclination suggests a similar degree of surface non-uniformity in the materials under investigation. A shift in the spectrum to the right along the real impedance axis may indicate a thicker or more impermeable passive layer, and thus poorer electrical contact between the powder particles, in addition to greater corrosion degradation.

**Figure 7:**
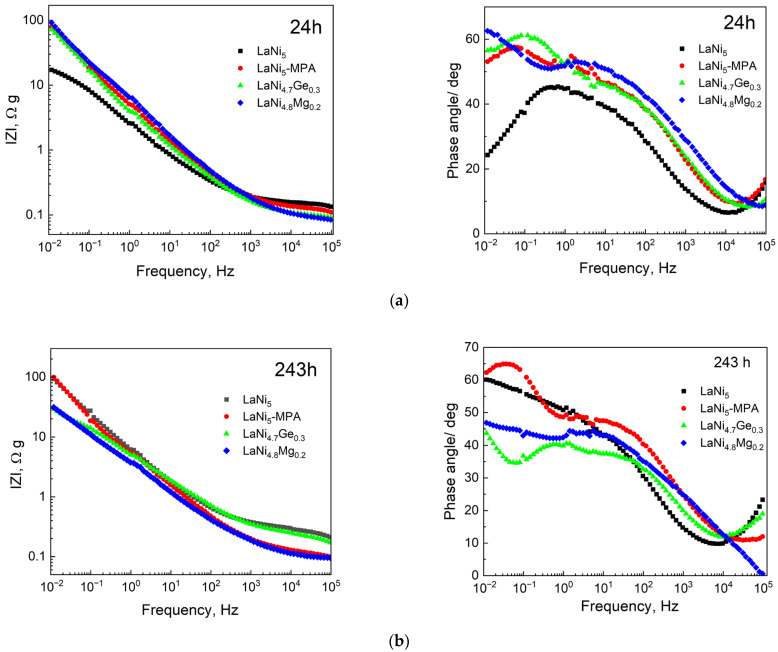
Bode plots of impedance spectra for the tested electrodes after (**a**) 24 and (**b**) 243 h of immersion in 6 M NaOH solution.

The evolution of the electrochemical interface was further investigated using Bode representations (Figure 7). After 24 h of immersion, all electrodes exhibit a similar slope in the impedance modulus (Z) at medium frequencies, suggesting that the fundamental porous architecture—primarily established during the powder compaction process—remains consistent regardless of the alloy composition. However, the vertical shifts in Z values indicate differences in the initially accessible active surface area, with the pristine LaNi_5_ showing the highest initial electrochemical activity.

The Bode phase plots provide deeper insight into the pore dynamics. The phase angle maxima for Ge- and Mg-substituted samples are shifted towards lower frequencies (0.1–0.01 Hz) compared to the base LaNi_5_. According to the Transmission Line Model (TLM), this shift correlates with an increased time constant, indicating higher tortuosity or deeper electrolyte penetration into the porous matrix.

After 243 h of immersion, a significant divergence in electrode behaviour is observed. The pristine LaNi_5_ and LaNi_5_-MPA samples show a substantial increase in IZI at low frequencies, which is consistent with the formation of a dense, passive layer of corrosion products that blocks the pore orifices. In contrast, the Mg- and Ge-modified electrodes exhibit the emergence of a characteristic phase angle minimum between 1 and 10 Hz. This feature provides evidence of a bimodal pore distribution or the development of a more permeable, porous corrosion layer.

These results confirm that while LaNi_5_ undergoes rapid self-passivation (leading to a sharp decline in corrosion rate but poor electrode kinetics), the Mg and Ge additions maintain the connectivity of the internal pore network. This ensures superior electrochemical stability and prevents surface deactivation, even though the material remains electrochemically active over extended periods. To facilitate the interpretation of the Bode plots presented in Figure 7, the main qualitative EIS features observed after 24 and 243 h immersion are summarised in Table 2. Since the investigated electrodes exhibit a porous and heterogeneous structure, the EIS analysis was performed qualitatively within the framework of the transmission line model (TLM), rather than by fitting simplified equivalent electrical circuits. Therefore, the interpretation is based primarily on characteristic changes in impedance modulus and phase angle behaviour.

**Figure 8:**
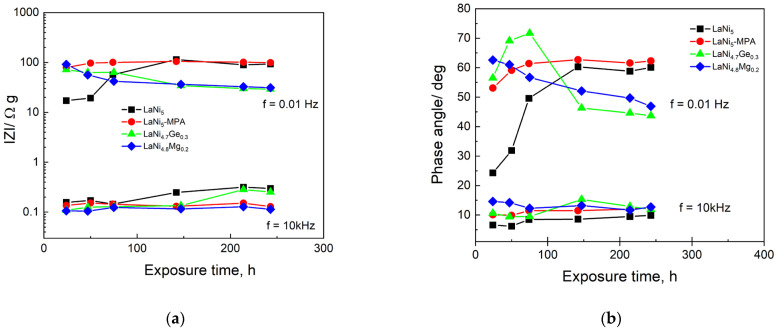
Values of impedance module |Z| (**a**) and phase angle (**b**) as a function of exposure time of investigated electrodes in 6 M NaOH solution.

Characteristic changes revealing the corrosion of alloys can be observed by analysing, for example, changes in the impedance modulus (Figure 8a) and phase angle (Figure 8b) as a function of exposure time in the electrolyte at a frequency of 10 kHz (morphology, surface roughness) and 0.01 Hz (corrosion resistance, diffusion resistance).

Both a high impedance modulus and a high phase angle at low frequencies in electrochemical impedance spectroscopy (EIS) indicate lower ionic conductivity and better corrosion resistance of the material. Analysis of the changes in the impedance modulus and phase angle at low frequencies reveals a marked slowing of corrosion processes for the LaNi_5_ reference electrode following prolonged (approximately 140 h) exposure in the solution. It is likely that more compact surface oxide structures form on the electrode, acting as a high-resistance barrier that impedes current flow at low frequencies. The opposite relationship can be observed for the electrodes partially substituted with germanium or magnesium, which exhibit better stability during the initial immersion period. Over time, both the impedance modulus and the low-frequency phase angle decrease, indicating progressive degradation of the oxide layer, which facilitates the penetration of ions into the electrode surface and a rise in the corrosion rate. A similar relationship between changes in the corrosion rate as a function of time for the researched electrodes can be observed in constant-current measurements (Figure 4).

The slight growth in the impedance modulus at 10 kHz for the original electrode and the electrode partially substituted with germanium, visible in Figure 8a, suggests the formation of a more continuous surface layer and/or a gradual increase in contact resistance between the particles of the powdered alloys. Interestingly, the LaNi_4.7_Ge_0.3_ electrode in the low-frequency range exhibits a lower impedance modulus compared to the reference LaNi_5_ and LaNi_5_-MPA electrodes after prolonged immersion in the electrolyte (>146 h). This behaviour indicates a more open pore network and better penetration of the electrolyte into the porous structure. Following prolonged contact with the electrolyte, there is also a sharp decrease in the phase angle for the LaNi_4.7_Ge_0.3_ electrode, which can be linked to the reorganisation of the pore structure and the development of an electrochemically active surface.

### 3.4. Surface Topography and Assessment of Electrode Corrosion Degradation After Hydrogenation Process

**Figure 9:**
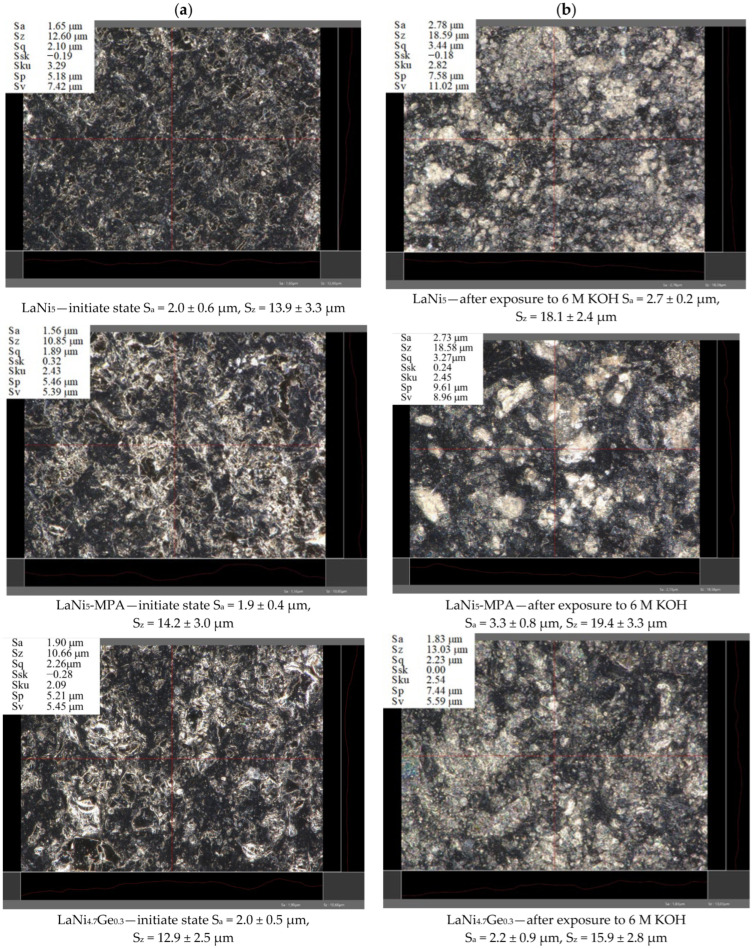

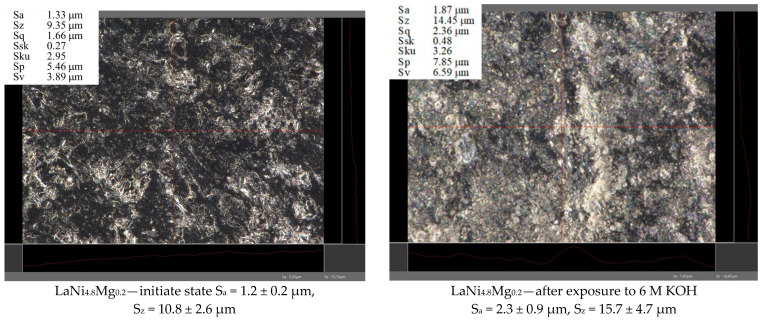
Micrographs of composite hydride electrode surfaces before (**a**) and after 243 h of immersion in 6 M NaOH solution (**b**). The image corresponds to an analysed area of 295.3 × 222.4 µm. The red lines indicate the profile lines used for surface roughness analysis.

A KEYENCE VHX-7000 microscope was used to assess the surface topography and roughness parameters S_a_ and S_z_ of the hydride electrodes. As can be seen from Figure 9, the action of concentrated alkaline electrolyte leads to a marked expansion of the surface and exposure of the metallic components of the composite materials under investigation.

**Figure 10:**
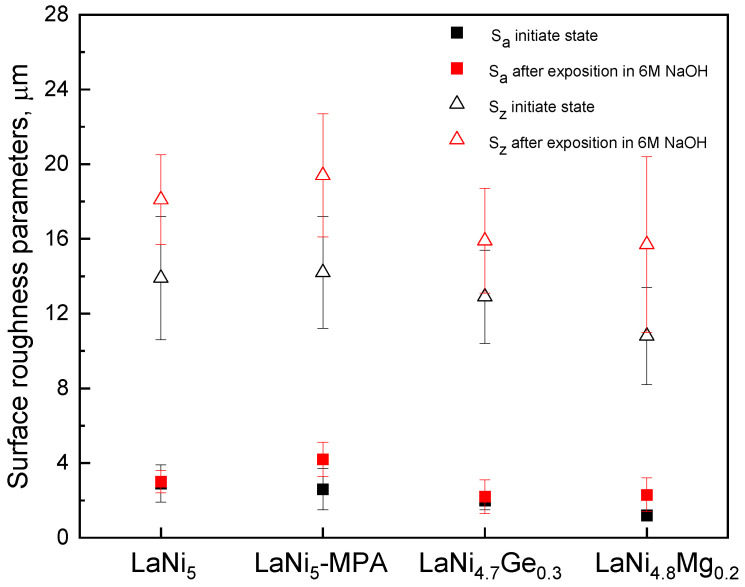
Changes in roughness parameters S_a_ and S_z_ before and after 243 h of exposure in 6 M NaOH.

An analysis of the results presented in Figure 10 reveals an increase in the surface roughness parameter S_z_ after 243 h of electrode exposure in 6 M NaOH. However, no significant differences were observed in the values of S_a_ and S_z_ for the individual electrodes, either before or after prolonged immersion in the electrolyte. Furthermore, the trend in changes in the impedance modulus and phase angle as a function of the immersion time of the electrodes in the corrosive solution at high frequencies (Figure 8) does not indicate any clear differences in the surface roughness of the individual electrodes.

**Figure 11:**
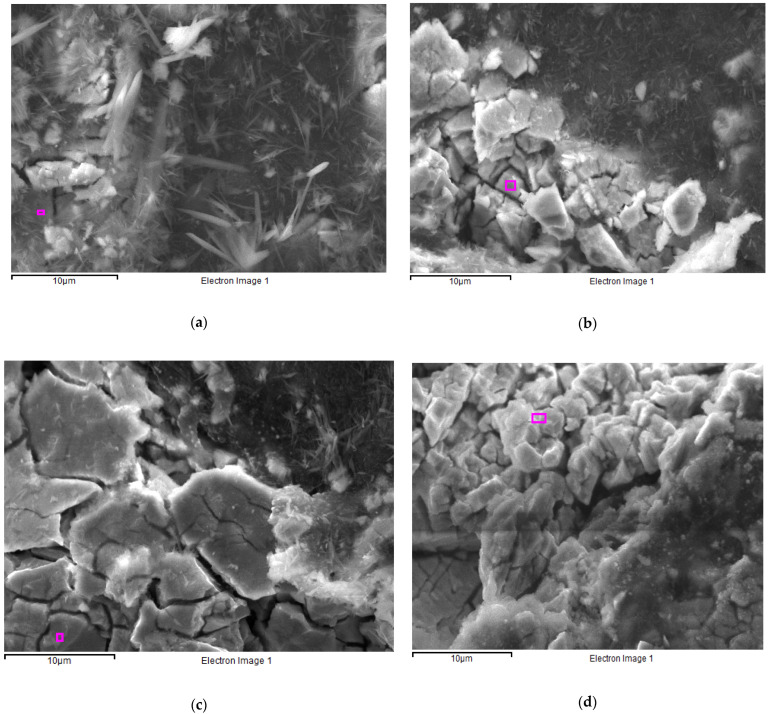
SEM images of composite hydride electrodes after activation and hydrogenation with indication of chemical composition analysis points: (**a**) LaNi_5_, (**b**) LaNi_5_-MPA, (**c**) LaNi_14.7_Ge_0.3_, (**d**) LaNi_14.8_Mg_0.2_. The pink squares mark one of the selected areas for EDS analysis.

The surface of the composite hydride electrodes was also examined by means of SEM imaging (Figure 11) following electrochemical activation (CV) and after 10 charge/discharge cycles at a current density of 185 mA g^−1^. The results of the quantitative point analysis of the alloy particle surface, obtained using EDS, are presented in Table 2.

In the case of the initial LaNi_5_ (Figure 11a) electrode, the very high oxygen content (~43 at.%) indicates intense oxidation processes and the formation of a thick layer of corrosion products on the surface. Furthermore, the low content of nickel (14 at.%) and lanthanum (3 at.%) suggests that the alloy surface is heavily covered by a layer of corrosion products. In the case of the other two electrodes, LaNi_5_-MPA (Figure 11b) and LaNi_4.7_Ge_0.3_ (Figure 11c), the oxygen content is significantly lower (~25 at.%), indicating a lower degree of surface oxidation. The higher content of nickel and lanthanum means that the corrosion product layer is thinner than in the case of the LaNi_5_ electrode. Analysis of the composition of the LaNi_4.8_Mg_0.2_ (Figure 11d) alloy particles shows that their surface is by far the least oxidised; the corrosion product layer either does not form at all or is very thin.

Furthermore, when analysing the ratio of the nickel to lanthanum content (Table 3), it can be seen that for the three studied electrodes (LaNi_5,_ LaNi_5_-MPA, LaNi_4.7_Ge_0.3_), this ratio is approximately consistent with the stoichiometry of these compounds. A corrosion layer containing both La and Ni is therefore formed, which is why the surface analysis yields an Ni/La ratio of ~5. The low oxygen content on the surface of the LaNi_4.8_Mg_0.2_ alloy particles and the high Ni/La ratio indicate surface enrichment of the electrode with nickel, which should result in improved kinetics of hydrogen evolution and absorption.

The differences in the long-term stability of the studied electrodes stem from distinct evolution mechanisms of their surface layers. For the pristine LaNi_5_ alloy, the high oxygen content (~43 at.%) and the sharp increase in the impedance modulus after 140 h indicate the formation of a thick, compact La(OH)_3_ layer. Through a self-sealing effect, this layer creates a high-resistance barrier that inhibits corrosion and facilitates hydrogen entrapment within the alloy bulk, as evidenced by the distinct anodic peak in the CV curves. In contrast, a different mechanism is observed for Ge and Mg-substituted electrodes. Despite their better initial stability, their protective layers undergo gradual degradation. For Ge-doped alloys, the amphoteric nature of germanium oxides [31] leads to their selective dissolution in KOH, resulting in pore network reorganisation and surface ‘opening’ (confirmed by the decrease in phase angle in EIS). For the Mg-substituted alloy, the low oxygen content in EDS suggests the absence of a continuous passive layer [32,33], promoting the exposure of fresh Ni active sites. In both cases, this leads to an increased active surface area and enhanced reaction kinetics (wave-like CV characteristics) at the expense of long-term corrosion resistance, which systematically deteriorates over time.

## 4. Conclusions

− LaNi_5−x_M_x_ alloys doped with magnesium or germanium retain a CaCu_5_-type hexagonal structure, with the addition of germanium increasing the unit cell volume, thereby facilitating hydrogen storage.
− All the electrodes have a heterogeneous and porous structure regardless of their chemical composition.
− Prolonged contact with the electrolyte increases the surface roughness (S_z_ parameter) of all the electrodes, although the differences between individual electrodes are insignificant.
− Electrodes based on the LaNi_5_ alloy form a high-resistance passive layer after an initial active corrosion phase (approx. 140 h), which effectively inhibits further corrosion processes. In contrast, the electrodes containing Ge or Mg exhibit the opposite trend—they are stable at the outset, but over time their oxide layer degrades (becomes compromised), which facilitates ion penetration and accelerates corrosion.
− The LaNi_5_ reference electrode has the most heavily oxidised surface (43 at.% oxygen).
− If higher power density and electrode capacity are the priority, a favourable solution is to partially replace the nickel in the LaNi_5_ alloy with magnesium or germanium. Nevertheless, if maximum service life (number of cycles) is key, the traditional LaNi_5_ alloy (or modified MPA) will perform better in aggressive alkaline environments.

## Figures and Tables

**Table 1:** Electrochemical parameters obtained from polarisation studies for the analysed samples.

LaNi_5_				
Time [h]	*E*_corr_ vs. SCE [V]	*R*_p_ [Ω g]	*β*_c_ (V/dec)	*I*_corr_ [A/g]
24	−0.98	4.1	0.10	0.010
74	−0.85	36	0.045	0.00050
214	−0.63	254	0.034	0.000060
243	−0.64	238	0.033	0.000060
LaNi_5_-MPA				
24	−0.83	54	0.050	0.00040
74	−0.75	116	0.051	0.00019
214	−0.69	129	0.066	0.00025
243	−0.69	130	0.06	0.00020
LaNi_4.7_Ge_0.3_				
24	−0.49	217	0.040	0.00008
74	−0.46	42	0.046	0.00048
214	−0.43	26	0.163	0.0027
243	−0.44	29	0.166	0.0025
LaNi_4.8_Mg_0.2_				
24	−0.18	588	0.050	0.000037
74	−0.26	131	0.028	0.000093
214	−0.44	45	0.126	0.0012
243	−0.44	39	0.126	0.0014

Note 1: Due to the high porosity of the electrodes, the corrosion current density (*I*_corr_) and polarisation resistance (*R*_p_) are normalised to the mass of the electrode (g) instead of the geometric surface area. Note 2: *R*_p_ and *I*_corr_ values were determined using the Linear Polarisation Resistance (LPR) method to ensure accuracy in the presence of strong passivation.

**Table 2:** Summary of qualitative EIS features and TLM-based surface interpretation.

Electrode Sample	Immersion Time	IZI at 0.01 Hz	Phase Angle Shape and Evolution	TLM Surface Interpretation
LaNi_5_	24 h	Low (~20 Ω g)	Broad peak shifted towards higher frequencies.	Presence of a thin, resistive surface oxide: shallow signal penetration.
	243 h	Significant increase (~100 Ω g)	No distinct peak, sharp rise in phase angle at low/mid frequencies.	Surface Passivation: formation of a thick, resistive corrosion product layer (e.g., La(OH)_3_) that increases the total line resistance.
LaNi_5_-MPA	24 h	High (~80 Ω g).	Maximum shifted towards lower frequencies (relative to LaNi_5_).	Pore Sealing: MPA increases the resistance within the pores (*R*_p_), slowing down the interfacial response.
	243 h	Stable (High ~100 Ω g)	Clear maximum at low frequencies.	Stable Barrier: stable passive layer maintains high impedance and a well-defined line response.
LaN _4.7_ Ge_0.3_	24 h	High (~70 Ω g)	Maximum shifted towards lower frequencies (similar to LaNi_5_-MPA).	Enhanced Passivation: Ge stabilises the surface film, increasing the capacitive response of the pore walls.
	243 h	Significant drop (~30 Ω g)	Presence of a minimum, plateau lowering.	Layer Instability: breakdown of the protective Ge-rich film, leading to “short-circuiting” of the TLM response.
LaNi_4.8_Mg_0.2_	24 h	High (~90 Ω g)	High phase angles, peak maximum potentially below 0.01 Hz.	Strong Barrier Effect: very slow charge transfer kinetics along the transmission line.
	243 h	Significant drop (~30 Ω g)	Flattening and lowering of the plateau.	Structural Degradation: Collapse of the Mg-modified surface layer, facilitating easier electrolyte access.

**Table 3:** Chemical composition analysis results of composite hydrogenation electrodes following electrochemical hydrogenation tests in 6 M NaOH (average values from measurement points).

Element (at.%)	LaNi_5_	LaNi_5_-MPA	LaNi_4.7_Ge_0.3_	LaNi_4.8_Mg_0.2_
C	26.32	18.15	28.09	10.38
O	43.3	25.38	25.21	3.98
F	2.14	-	-	-
Na	11.30	4.05	12.51	1.73
Ni	13.94	43.12	26.46	73.67
La	3.01	9.3	5.58	10.24
Ge	-	-	2.16	-
Mg	-	-	-	not detected

## Data Availability

The original contributions presented in this study are included in the article. Further inquiries can be directed to the corresponding author.
